# Aβ and tau prions feature in the neuropathogenesis of Down syndrome

**DOI:** 10.1073/pnas.2212954119

**Published:** 2022-11-07

**Authors:** Carlo Condello, Alison M. Maxwell, Erika Castillo, Atsushi Aoyagi, Caroline Graff, Martin Ingelsson, Lars Lannfelt, Thomas D. Bird, C. Dirk Keene, William W. Seeley, Daniel P. Perl, Elizabeth Head, Stanley B. Prusiner

**Affiliations:** ^a^Institute for Neurodegenerative Diseases, Weill Institute for Neurosciences, University of California, San Francisco, CA 94158;; ^b^Department of Neurology, Weill Institute for Neurosciences, University of California, San Francisco, CA 94158;; ^c^Department of Pharmaceutical Chemistry, University of California, San Francisco, CA 94158;; ^d^Daiichi Sankyo Co., Ltd., Tokyo, Japan 103-8426;; ^e^Department of Neurobiology, Care Sciences and Society, Karolinska Institute, Solna, Sweden 171 77;; ^f^Unit for Hereditary Dementias, Karolinska University Hospital, Solna, Sweden SE-171 76;; ^g^Department of Public Health and Caring Sciences/Geriatrics, Uppsala University, Uppsala, Sweden SE-751 05;; ^h^Krembil Brain Institute, University Health Network, Toronto, ON, Canada M5T 2S8;; ^i^Department of Medicine and Tanz Centre for Research In Neurodegenerative Diseases, University of Toronto, Toronto, ON, Canada M5S 1A8;; ^j^Department of Medicine, Division of Medical Genetics, University of Washington, Seattle, WA 98195;; ^k^Department of Neurology, University of Washington, Seattle, WA 98195;; ^l^Department of Laboratory Medicine and Pathology, University of Washington School of Medicine, Seattle, WA 98115;; ^m^Department of Pathology, University of California, San Francisco, CA 94143;; ^n^Department of Pathology (Neuropathology), F. Edward Hébert School of Medicine, Uniformed Services University of the Health Sciences, Bethesda, MD 20814;; ^o^Department of Pathology and Laboratory Medicine, University of California, Irvine, CA 92697;; ^p^Department of Biochemistry and Biophysics, University of California, San Francisco, CA 94158

**Keywords:** Down syndrome, Aβ, tau, prions, cellular bioassays

## Abstract

Down syndrome (DS) is caused by the triplication of chromosome 21 and is the most common chromosomal disorder in humans. Those individuals with DS who live beyond age 40 y develop a progressive dementia that is similar to Alzheimer’s disease (AD). Both DS and AD brains exhibit numerous extracellular amyloid plaques composed of Aβ and intracellular neurofibrillary tangles composed of tau. Since AD is a double-prion disorder, we asked if both Aβ and tau prions feature in DS. Frozen brains from people with DS, familial AD (fAD), sporadic AD (sAD), and age-matched controls were procured from brain biorepositories. We selectively precipitated Aβ and tau prions from DS brain homogenates and measured the number of prions using cellular bioassays. In brain extracts from 28 deceased donors with DS, ranging in age from 19 to 65 y, we found nearly all DS brains had readily measurable levels of Aβ and tau prions. In a cross-sectional analysis of DS donor age at death, we found that the levels of Aβ and tau prions increased with age. In contrast to DS brains, the levels of Aβ and tau prions in the brains of 37 fAD and sAD donors decreased as a function of age at death. Whether DS is an ideal model for assessing the efficacy of putative AD therapeutics remains to be determined.

## Significance

Approximately 5.4 million people worldwide have Down syndrome (DS), which is caused by trisomy of chromosome 21 (Chr21). The *APP* gene is one of approximately 250 protein-coding genes located on Chr21, and its duplication is associated with elevated Aβ production and increased incidence of Alzheimer’s disease (AD) neuropathology in most aged individuals with DS. Since AD brains have plaques composed of Aβ prions and neurofibrillary tangles composed of tau prions, we asked if DS brains have both Aβ and tau prions. We found that the age-dependent kinetics of Aβ and tau prions are distinct in DS and could even be detected in a 19-y-old individual. Whether DS is an ideal model for assessing efficacy of putative AD therapeutics remains unknown.

More than a century ago, both Down syndrome (DS) (1) and Alzheimer’s disease (AD) were first described (2). In the United States, there are ∼400,000 people with DS and ∼5.4 million worldwide (3). DS, which is caused by triplication of chromosome 21(Chr21) (4), results in a shortened life expectancy ranging from 20 to 70 y. Intellectual disability is almost universal in people with DS; the average intelligence quotient for individuals with DS is ∼50 (5). Co-occurring illnesses include obstructive sleep apnea, otitis media, congenital heart disease, gastrointestinal atresia, thyroid disease, and seizure disorders (6).

Over the past six decades, longevity of people with DS has markedly increased and is thought to be due to improved access to and efficacy of surgeries for congenital heart disease and medical treatments for respiratory infections (7). Though medical advances have reduced institutionalization and increased integration into society, it is unknown if such changes have reduced the prevalence of AD in DS.

For many years, AD was considered a presenile dementia with plaques and tangles in people younger than 65 y, while people older than 65 y with plaques and tangles were diagnosed with senile dementia. In 1982, Terry et al. (8) argued that the presenile dementia called AD was indistinguishable from the plaques and tangles of most demented older people and should be merged and called AD. At about the same time, purified amyloid fibrils recovered from brains of deceased people with AD as well as DS were found to consist of a unique amino acid sequence (later called Aβ) (9), which was subsequently found in AD plaques (10).

Contemporaneously with the discovery of the Aβ peptide, immunostaining identified that neurofibrillary tangles (NFTs) contained the tau protein (11). Four decades earlier, Jervis (12) reported that deceased people with DS have both senile plaques and NFTs, based on Bielschowsky silver staining in fixed brain sections. Aβ plaques and tau NFTs are considered a common neuropathological feature in most individuals with DS older than 40 y (13). The anatomical distribution and biochemical properties of Aβ plaques and NFTs are similar to those of AD (14, 15), which are thought to contribute to progressive dementia and related biomarker changes in approximately two-thirds of aged people with DS (16–20). Given these comparable molecular and clinical traits, we hypothesized that studying aged people with DS might afford a new perspective in unraveling the molecular pathogenesis of some neurodegenerative diseases (NDs) caused by prions.

In the last two decades, numerous studies have shown that both Aβ and tau proteins adopt pathogenic, self-propagating conformations characteristic of prions (21–26). Prions induce the misfolding of additional copies of the naïve protein (e.g., Aβ or tau) in a self-perpetuating process that spreads within and between neural cells (what we call infectivity at the cellular level). To be clear, no definitive evidence exists to suggest that either AD or DS are communicable disorders; this is in contrast to Creutzfeldt-Jakob disease and kuru, both of which have shown to be caused by readily transmissible prion proteins (PrPs) (27–29). Importantly, self-propagating forms of unrelated proteins in yeast and other fungi were found to have beneficial, rather than pathological, roles in these organisms (30). In mammals, proteins involved in memory formation and synapse plasticity adopt a prion conformation as part of their biochemical function in neurons (31–33). Thus, prions mediate diverse processes in organisms separated by hundreds of millions of years of evolution.

The first experimental transmission of prions from people with DS and AD to marmosets was reported by Ridley et al. (34). They transmitted central nervous system disease from the brain tissue of two deceased male donors with DS (ages 35 and 64 y) to six marmosets by intracerebral inoculation of brain homogenates. Although the inoculated marmosets did not develop signs of neurological dysfunction, they did exhibit deposits of the Aβ peptide and were killed humanely due to medical welfare issues. The disease in marmosets had an incubation period of 4 to 8 y. Aβ deposits were identified by Congo red birefringence, thioflavin staining, and anti-Aβ immunostaining. Notably, no NFTs were found by silver staining or immunostaining for tau, and no Aβ aggregates were identified in uninoculated marmosets that lived up to 19 y of age.

To shorten the incubation period and expand the scale of such inoculation studies, investigators inoculated transgenic (Tg) mice with human AD brain extracts. However, this approach still required incubation periods of several months to a year, as well as separate mouse models to detect the presence of Aβ or tau prions in a particular brain extract. Based on a rapid human cell bioassay previously developed for the measurement of tau prions (35), we built parallel cell lines to measure α-synuclein, Aβ, and tau prions on the same platform. With this approach, we measured prion infectivity found in eight different NDs: AD, cerebral amyloid angiopathy, progressive supranuclear palsy, corticobasal degeneration, Pick’s disease, chronic traumatic encephalopathy, multiple system atrophy, and dementia with Lewy bodies (26, 36–38). Here, we applied a similar approach to measuring Aβ and tau prions in brains from people with DS using the human cell bioassays.

## Results

### Aβ and Tau Prions in Brains of People with DS.

To measure Aβ and tau prions in brain tissues from deceased donors, we used human embryonic kidney 293T (HEK293T) cells expressing the yellow fluorescent protein (YFP) fused to either tau or Aβ (*Materials and Methods*). Upon prion infection of HEK293T cells, the accumulation of YFP–prion aggregates could be measured as fluorescent puncta (26, 39).

**Fig. 1.**
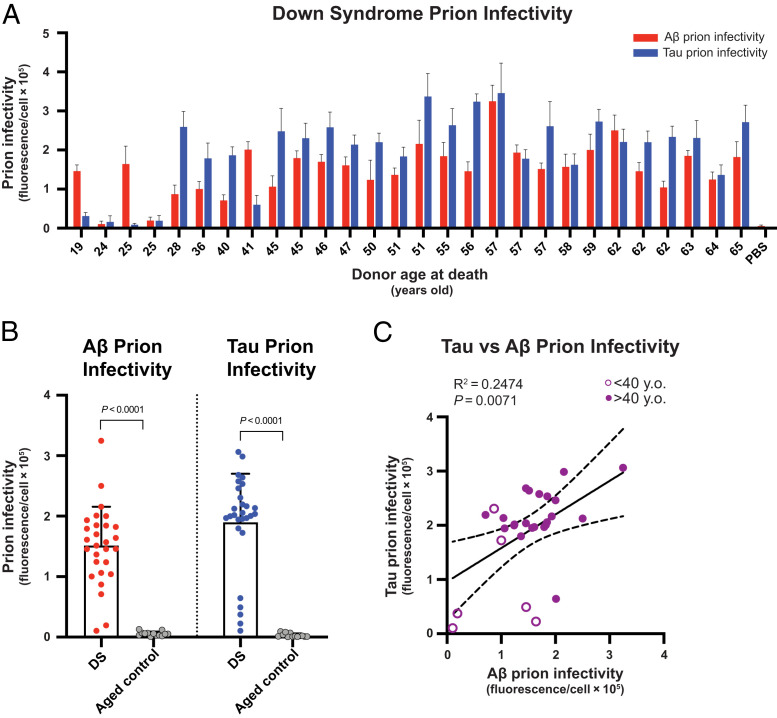
Cell bioassays detect Aβ and tau prions in DS brain samples. (*A*) Diluted (0.03×) PTA extracts from frozen brain samples of adults with DS were added to HEK293T cells expressing YFP–Aβ42 or tau–YFP to measure Aβ and tau prion infectivity, respectively. Cell-based prion infectivity measurements plotted as a function of the donor age at death. Data are presented as the mean and SD of four technical replicates per individual subject per assay. PBS control refers to the vehicle buffer with lipofectamine that is used in the cell infection protocol. (*B*) Bar graphs showing group comparison of Aβ and tau prion infectivity for DS and cognitively neurotypical, age-matched controls. Data are presented as the mean and SD of all samples per group. Aβ prion infectivity values are as follows: 1) DS = 151,420 arbitrary units (a.u.) ± 64,311; 2) aged control = 5,522 a.u. ± 3,594. Tau prion infectivity values are as follows: 1) DS = 189,912 a.u. ± 80,154; 2) aged control = 2,980 a.u. ± 3,131. Student’s *t* test was used to assess statistical significance compared with aged controls. (*C*) Tau prion infectivity was plotted as a function of Aβ prion infectivity for each sample with DS, and a linear regression was performed. Individuals with DS who were ≥40 y old (filled circles) and those who were younger than 40 y (open circles) were plotted together. y.o., years old.

To investigate the unique etiological parallels between DS and AD, we measured the levels of Aβ and tau prions in frontal brain cortices of 28 deceased patients with DS who ranged from age 19 to 65 y (median age, 51 y; *SI Appendix*, Table S1) and 14 deceased control cases with no cognitive impairment, who ranged in age from 27 to 70 y (median age, 48 y; *SI Appendix*, Table S1). Sodium phosphotungstic acid (PTA) was used to selectively precipitate prions from brain homogenates (40). To establish the conditions for performing the bioassays, we prepared a small dilution series of a subset of brain extracts with which to infect each cell line (*SI Appendix*, Fig. S1). With a few exceptions in the younger cases, we measured robust Aβ infectivity (*P <* 0.0001) and tau prion infectivity (*P <* 0.0001) in nearly all DS samples compared with a negative control (Fig. 1 *A* and *B*). Interestingly, two of the youngest cases in our cohort showed nominal tau prion infectivity but did exhibit robust Aβ prion levels, similar to those in older cases with DS. Such Aβ prion abundance in these young individuals was present despite only minimal AD neuropathological burden in corresponding fixed sections (*SI Appendix*, Fig. S2 *A* and *B*) and biochemical measurements in extracts from frozen tissue (*SI Appendix*, Fig. S2 *D* and *E*). Consistent with recent work (41, 42), this finding suggests that Aβ prions likely emerge long before the development of mature Aβ plaques, which are typically found at age 40 y or older for people with DS (43). Overall, the mean levels of Aβ prions in the DS cohort were 27 times greater (*P <* 0.0001) than the cognitively neurotypical age-matched controls (Fig. 1*B*). Additionally, the mean levels of tau prions in the DS cohort were 63 times greater (*P <* 0.0001) than the cognitively neurotypical age-matched controls (Fig. 1*B*). We also found that the abundance of tau prions had a modest linear correlation with the abundance of Aβ prions (*R*^2^ = 0.2474; *P* = 0.0071) in people with DS (Fig. 1*C*), which was consistent with the AD neuropathology scores (*SI Appendix*, Fig. S2*C*) and biochemical measurements (*SI Appendix*, Fig. S2*F*). Our findings demonstrate that DS is also a double-prion disorder, like AD, featuring both Aβ and tau prions.

### Correlations Between Aβ and Tau Prions in Brains with Familial AD and Sporadic AD.

Next, we compared prion infectivity levels in similarly aged people with DS and with AD. We asked if linear relationships between Aβ and tau prion infectivity might exist in two different, etiologically distinct disorders. We evaluated postmortem brain samples from a cohort of 26 early-onset familial AD (fAD) cases ranging in age from 37 to 78 y (median age, 58 y; *SI Appendix*, Table S2). We procured brain samples from deceased donors with fAD bearing autosomal-dominant mutations in the genes *APP*, *PSEN1*, or *PSEN2*. We also included samples from 17 donors with sporadic AD (sAD) ranging in age from 59 to 88 y (median age, 70 y; *SI Appendix*, Table S2) and 10 control donors with no cognitive impairment and ranging in age from 62 to 88 y (median age, 67 y; *SI Appendix*, Table S2).

**Fig. 2.**
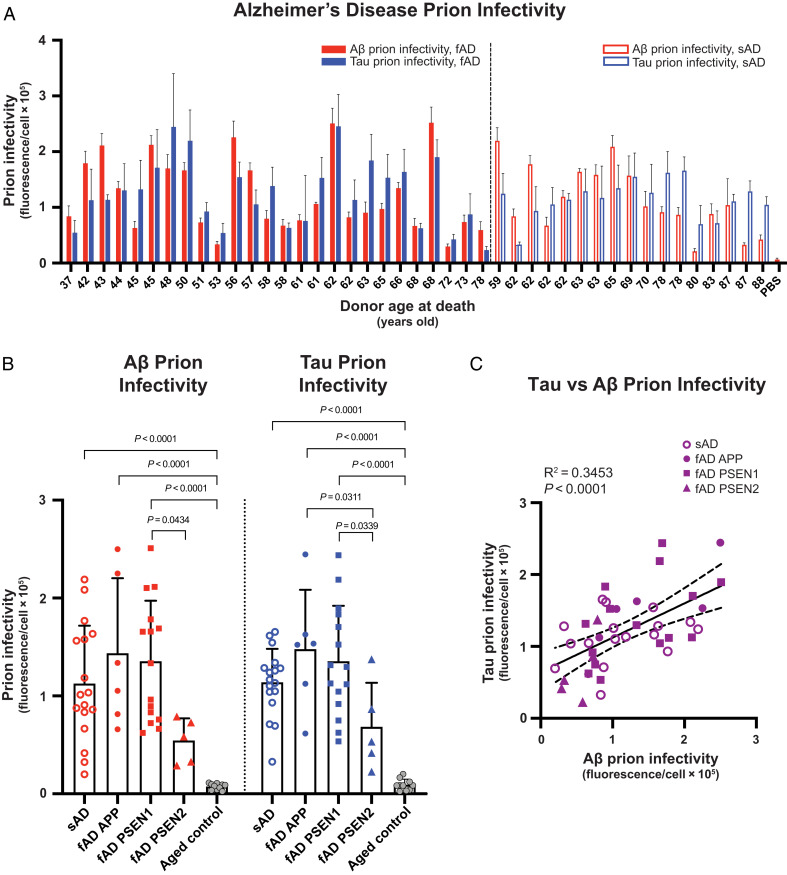
Cell bioassays detect Aβ and tau prions in fAD and sAD brain samples. (*A*) Diluted (0.03×) PTA extracts from frozen fAD and sAD brain samples were added to HEK293T cells expressing YFP–Aβ42 or tau–YFP to measure Aβ and tau prion infectivity, respectively. Cell-based prion infectivity measurements plotted as a function of the donor age at death. Data are presented as the mean and SD of four technical replicates per individual subject per assay. PBS control refers to the vehicle buffer with lipofectamine that is used in the cell infection protocol. (*B*) Bar graphs showing group comparison of Aβ and tau prion infectivity for fAD, sAD, and cognitively normal, age-matched controls. Data are presented as the mean and SD of all samples per group. Aβ prion infectivity values are as follows: 1) sAD = 112,557 arbitrary units (a.u.) ± 59,276; 2) fAD APP = 143,570 a.u. ± 76,647; 3) fAD PSEN1 = 135,387 a.u. ± 61,832; 4) fAD PSEN2 = 54,358 a.u. ± 22,782; and 5) aged control = 7,439 a.u. ± 3,143. Tau prion infectivity values are as follows: 1) sAD = 113,925 a.u. ± 34,152; 2) fAD APP = 147,827 a.u. ± 60,521; 3) fAD PSEN1 = 135,374 a.u. ± 56,701; 4) PSEN2 = 68,340 a.u. ± 45,068; and 5) aged control = 9,254 a.u. ± 5,717. A two-way ANOVA with Tukey’s multiple comparison test was used to assess statistical significance compared with aged controls. Comparisons between all groups were made, but only comparisons that reached statistical significance are annotated on the graph. (*C*) Tau prion infectivity was plotted as a function of Aβ prion infectivity for each fAD and sAD donor, and a linear regression was performed.

Our cell bioassays demonstrated that the brain samples with fAD and sAD contained appreciable Aβ and tau prion infectivity levels compared with a negative control (Fig. 2*A*). While the prion levels in brain samples with AD were heterogeneous across the age range examined, the overall mean values for Aβ prion infectivity in the sAD (*P <* 0.0001), fAD APP (*P <* 0.0001), and fAD PSEN1 (*P <* 0.0001) cohorts were at least 15 times greater than the cognitively neurotypical age-matched controls (Fig. 2*B*). Similarly, the mean values for tau prion infectivity in the sAD (*P <* 0.0001), fAD APP (*P <* 0.0001), and fAD PSEN1 (*P <* 0.0001) cohorts were at least 12 times greater (*P* < 0.0001) than the cognitively neurotypical age-matched controls (Fig. 2*B*). No statistical difference in Aβ and tau prion infectivity levels was found when comparing sAD, fAD APP, and fAD PSEN1 with each other. Curiously, the mean values for Aβ and tau prion infectivity in the fAD PSEN2 cohort exhibited a marked increase compared with the controls, yet they did not reach statistical significance using a two-way ANOVA. In addition, we found that the abundance of tau prions was linearly correlated with the abundance of Aβ prions (*R*^2^ = 0.3453; *P* < 0.0001) in all people with AD (Fig. 2*C*). Taken together, these data indicate three important findings: First, bioactive Aβ and tau prions persisted in diseased brains at the time of death, which is consistent with our earlier findings (26). Second, these data provide unequivocal evidence that DS, fAD, and sAD all produce Aβ and tau prions, but each arises from distinct etiological pathways. Third, the abundance of Aβ prions seems to govern the level of tau prions in both DS as well as AD.

### Increased Abundance of Aβ and Tau Prions in Older People with DS.

**Fig. 3.**
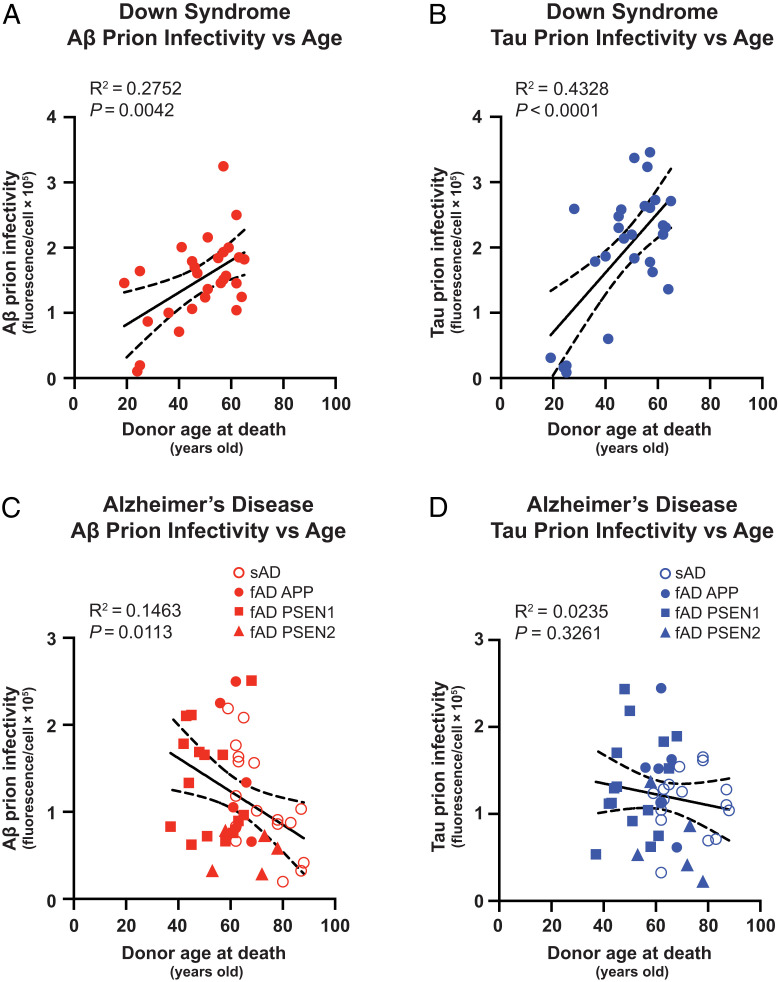
Aβ and tau prion abundance increases with age in DS but not in AD. (*A* and *B*) Aβ and tau prion infectivity in DS brain samples plotted as a function of donor age at death. (*C* and *D*) Aβ and tau prion infectivity in fAD (filled symbols) and sAD (open symbols) brain samples plotted as a function of donor age at death. Linear regression was performed in all panels.

Because of the overall similarities in prion infectivity between DS and AD, we questioned if the abundance of Aβ and tau prions was lower in longer-lived individuals with DS, similar to prior observations we reported for a large cohort of donors with fAD and sAD (26). We plotted Aβ and tau prion infectivity as a function of the donor’s age at death and performed a linear regression analysis. Unexpectedly, we found significant trends of increased Aβ prions (*R*^2^ = 0.2752; *P* = 0.0042) and tau prions (*R*^2^ = 0.4328; *P* < 0.0001) in people with DS who lived longer (Fig. 3 *A* and *B*), which was consistent with the neuropathological and biochemical measurements of Aβ and tau proteins (*SI Appendix*, Fig. S2). This result contrasted starkly with the same analysis performed on the fAD and sAD prion infectivity data (Fig. 3 *C* and *D*). Moreover, fAD and sAD samples were plotted together because of the gross similarities in abundance of Aβ and tau prions, and meta-analyses of many longitudinal biomarker studies argue that fAD and sAD share a common sequence of pathological events (44, 45). We observed that Aβ prions exhibited a significant decrease (*R*^2^ = 0.1463; *P* = 0.0113) in abundance with increasing age at death (Fig. 3*C*). While we did not observe any significant trend for tau prion infectivity with age in this study (*R*^2^ = 0.0235; *P* = 0.3261), we reported a negative correlation of tau prion abundance and age at death in a larger AD cohort that included samples from much older donors (26). We repeated the regression analysis on only the sAD cohort and found it was driving the negative trend (*R*^2^ = 0.4632; *P* = 0.0026) in Aβ prion infectivity levels plotted as a function of age at death (*SI Appendix*, Fig. S3*A*); in the regression analysis of only the fAD cohort, Aβ prion infectivity levels plotted as a function of age at death exhibited a subtle but insignificant negative trend (*SI Appendix*, Fig. S3*C*). There was no change in the statistics of the regression analysis for tau prion infectivity when plotting data from only sAD (*R*^2^ = 0.0031; *P* = 0.8318; *SI Appendix*, Fig. S3*B*) or fAD (*R*^2^ = 0.0306; *P* = 0.3930; *SI Appendix*, Fig. S3*D*).

To study these relationships in an overlapping age range, we repeated the regression analyses using only a subset of DS and AD samples from donors who died between 40 and 60 y of age (*SI Appendix*, Fig. S4). We observed positive, albeit insignificant, trends for Aβ prions (*R*^2^ = 0.1772; *P* = 0.1044) and tau prions (*R*^2^ = 0.1965; *P =* 0.0855) in people with DS who lived longer (*SI Appendix*, Fig. S4 *A* and *B*). We observed slightly negative but insignificant trends for Aβ prions (*R*^2^ = 0.0358; *P* = 0.5828) and tau prions (*R*^2^ = 0.0582; *P =* 0.4059) in people with AD who lived longer (*SI Appendix*, Fig. S4 *C* and *D*). While we found that all trends in this age subgroup were consistent with the original analyses, including DS and AD samples from the entire age range (Fig. 3), larger studies in the future will further explore the relationships of prion infectivity, age at death, and other relevant biological variables. For example, like in AD, DS carriers of the *APOEε4* risk allele have increased risk for AD and mortality (46, 47) and more severe neuropathology (48, 49), but we did not observe any trends in our data from the cohort of 28 individuals. Similar to our prior study of prion levels in long-lived AD donors (26), we performed a battery of immunochemical assays for APP, Aβ, and tau proteins in the soluble and insoluble brain fractions but did not observe any obvious trends with donor age at death or correlation to abundance of prion infectivity (*SI Appendix*, Figs. S5 and S6). Because of interdonor variability, we speculate that potential relationships may exist with greater sampling. Thus, work with larger DS cohorts is underway to examine the relationships of Aβ/tau protein levels and genetic risk factors (e.g., *APOEε4*) on the kinetics and severity of Aβ and tau prion accumulation, especially in young individuals. In summary, our data argue that the AD and DS prions represent different strains.

## Discussion

Our findings demonstrate that the brains of people with DS feature both Aβ and tau prions, which appear to be indistinguishable from the two prions that accumulate in both the sporadic and familial forms of AD. Importantly, DS is neither sporadic nor inherited, but it is a genetic disease caused by complete or partial triplication of Chr21. In trisomic individuals who bear an extra copy of the *APP* gene, the overexpression of wild-type (WT) APP results in increased levels of Aβ prions. Conversely, partial trisomy lacking triplication of *APP* does not lead to the neuropathologic changes of AD (50, 51). While we have previously reported that Aβ prions in the absence of tau prions result in cerebral amyloid angiopathy (26), this is not the case for DS. We found both Aβ and tau prions in nearly all of the brains of our DS cohort. In agreement with others, we propose that research in people with DS may help clarify sAD pathogenesis, given that both neuropathology and prion infectivity closely resemble that found in sAD, the predominant form of AD.

Notably, some individuals with DS exhibit many co-occurring conditions, including heart defects, obesity, diabetes, and progeria. How these conditions in people with DS modify the central nervous system dysfunction in the aging DS brain is unclear. There is evidence from mouse models that triplication of some Chr21 homologs increases Aβ deposition independently of an extra *APP* copy (52); conversely, *APP* duplication alone is sufficient to cause AD (53). *APP* duplications in DS provide an interesting comparison to Tg(APP) mice, which also overexpress human APP and Aβ. However, we note that plaques only form in Tg mice bearing familial mutations in *APP* and not WT *APP*; efforts to knock-in the WT human *APP* allele or humanize the Aβ peptide sequence within rodent *App* do not lead to plaque formation in the lifespan of a mouse (54, 55). Moreover, while the first generation of DS mouse models, segmental trisomy of mouse Chr16 (e.g., Ts65Dn) (56, 57), do replicate many neurodevelopmental phenotypes and present age-related neurodegeneration, they do not produce robust Aβ pathology in aged mice (58). One caveat of the Ts65Dn model is that it duplicates genes not present on human Chr21. To avoid this, new models employing transchromosomic (Tc) techniques in mice and rats have been developed in which the long arm of human Chr21 is cloned into the rodent genome. Despite this advancement, there is still a lack of Aβ plaque formation during the Tc(Chr21) rodent lifespan (59, 60). Whether or not Aβ prions could be measured in Tc(Chr21) rodents using cellular bioassays remains to be determined. Nevertheless, these findings suggest that the formation of Aβ and tau prions as well as AD neuropathology resulting from overexpression of WT human APP is a uniquely human condition. These findings make it critical to use human brain samples wherever possible to investigate the molecular pathogenesis of DS.

Effects of Aβ concentration on the formation of Aβ prion strains may be amenable to study in both rodents and humans. In prior work, we demonstrated that the brain concentrations of APP, Aβ40, and Aβ42 proteins in long-lived people with AD trended significantly lower (*P* < 0.005) compared with people who died much younger (26). This matches the lower Aβ prion infectivity observed with cell bioassays in those same people (26). If such a trend was present from a young age, it might indicate that low APP expression over the lifespan contributes to an Aβ prion strain that is less pathogenic or slower to accumulate and contributes to longevity. Interestingly, using amyloid strain-sensitive dyes and spectral imaging methods in fixed tissues (61), we found that the conformation of Aβ plaques in aged individuals with DS and advanced neuropathology showed a distinct conformational strain phenotype, compared with sAD (62). While the relationship between amyloid plaque conformation and Aβ prion infectivity remains to be determined, there is growing evidence that supports the notion that pathogenic Aβ and tau species in DS may differ from fAD and sAD in ways not appreciated with traditional histological and biochemical measurements.

Our prion bioassays allow for measurement of both Aβ and tau prions in DS rather than inert protein deposits. The finding that Aβ and tau prions are positively correlated in DS and AD agrees well with genetic and experimental studies arguing that Aβ prions arise early in AD pathogenesis and that these prions initiate subsequent tau prion formation (63–65). Consistent with this notion, we found that samples from two of the youngest individuals with DS in our study (19 and 25 y old) exhibited robust levels of Aβ prions but insignificant levels of tau prions; in adjacent formalin-fixed sections, we found that these donors had low levels of plaques and tangles (*SI Appendix*, Fig. S2). In contrast, we have not found any brains with DS or AD that have readily detectable levels of tau prions accompanied by marginal levels of Aβ prions. Indeed, our studies of primary tauopathies such as progressive supranuclear palsy and corticobasal degeneration have failed to show any detectable Aβ prions (26). To our knowledge, individuals with DS do not present with only NFTs in the absence of amyloid plaques (15). This finding is consistent with our view that Aβ prions initiate formation of tau NFTs in the vast majority of people with AD as well as DS.

Indeed, the cellular bioassays provide a functional readout of self-replicative proteins but do not provide the biophysical or structural characteristics of a given prion. It will be important for future mechanistic and drug discovery research to more precisely understand the molecular features of Aβ and tau prions in DS and AD. For example, Aβ peptides assemble into aggregates, which are called oligomers when the aggregate size is less than ∼50 peptides (66). A multitude of studies on human brain samples have reported the existence of soluble Aβ oligomers ranging in size, including dimers, trimers, and tetramers (67). Oligomer size has also been found to correlate inversely with cellular toxicity (68, 69). Moreover, the abundance of Aβ oligomers correlates well with the progression of cognitive deficits (70–72) and can differentiate patients with AD from nondemented people with comparable amyloid plaque burden (73). Extensive studies of Aβ oligomers in DS are lacking, but a few reports indicate an early (preplaque accumulation) and persistent increase of Aβ oligomers in aging DS people (74, 75). This is consistent with our data showing abundant Aβ prions in young people with DS with little to no amyloid plaque pathology. To our knowledge, there are no reports describing the characterization of tau oligomers in the brains of DS donors. Whether Aβ or tau multimer size correlates with prion infectivity and pathological deposition remains to be determined. By quantifying the oligomeric distribution and concentration, it should be possible to establish a relationship between the number of proteins in an oligomer and its prion infectivity (i.e., the particle to infectivity ratio [P/I]). For example, the P/I is ∼5,000 for the scrapie PrP isoform (76).

DS reveals a new vista of prion biology where trisomy of Chr21 results in increased Aβ production from an early age and leads to the formation of Aβ prions (77–80). It will be important to determine if this phenomenon occurs in all people with DS or a subset and to establish the earliest ages of prion detection. Despite the extraordinary contrast in etiologies between two genetic forms of Aβ prion diseases, one of which is nonheritable (DS) and the other heritable (fAD), both DS and fAD lead to a convergent neuropathogenic phenotype. Notably, by including sAD with fAD and DS, these three double-prion diseases are the most frequent neurodegenerative conditions worldwide, in which Aβ prions stimulate tau prions to cause neurodegeneration. Moreover, DS joins the expanding spectrum of NDs known to be caused by pathogenic prions (Table 1). Indeed, PrP prions cause Creutzfeldt-Jakob disease and kuru and can manifest in sporadic, heritable, and communicable disorders. While the other NDs can be sporadic or heritable, there is little evidence that Aβ, tau, or α-synuclein prions are communicable or spread by iatrogenic transmission (102–105). However, Aβ, tau, or α-synuclein prions extracted from donor brains of each disease can be transmitted to experimental animals or cultured human cells. These transmission models have enabled investigations of prion disease mechanisms and preclinical testing of novel therapeutic candidates.

**Table 1.** Experimental transmission of prions derived from human brain extracts to animals or cultured cells

**Disease etiology**	**Sporadic**	**Heritable**	**Communicable**	**Experimental prion transmission**				**Selected citations: Experimental transmission in animal and human cell bioassays**
				**Aβ**	**Tau**	**α-Syn**	**PrP**	
Down syndrome	**+**	**−**	**−**	**+**	**+**	n.d.	n.d.	Aβ and tau (81)
Sporadic Alzheimer’s disease	**+**	**−**	**−**	**+**	**+**	**−**	n.d.	Aβ (22, 25, 26, 81, 82); tau (26, 81, 83); α-syn (26)
Familial Alzheimer’s disease	**−**	**+**	**−**	**+**	**+**	**−**	n.d.	Aβ (25, 26, 81); tau (26, 81); α-syn (26)
Sporadic cerebral amyloid angiopathy	**+**	**−**	**−**	**+**	**−**	**−**	n.d.	Aβ, tau, and α-syn (26)
Familial cerebral amyloid angiopathy	**−**	**+**	**−**	**+**	**−**	**−**	n.d.	Aβ, tau, and α-syn (26)
Progressive supranuclear palsy	**+**	**+**	**−**	**−**	**+**	**−**	n.d.	Aβ (26); tau (26, 35, 37, 83, 84); α-syn (26)
Corticobasal degeneration	**+**	**+**	**−**	**−**	**+**	**−**	n.d.	Aβ (26); tau (26, 35, 37, 83–85); α-syn (26)
Argyrophilic grain disease	**+**	**+**	**−**	n.d.	**+**	n.d.	n.d.	Tau (35, 37, 83–85)
Pick’s disease	**+**	**+**	**−**	n.d.	**+**	n.d.	n.d.	Tau (26, 35, 37, 83–85)
Chronic traumatic encephalopathy	**+**	**+**	**−**	n.d.	**+**	n.d.	n.d.	Tau (37, 86)
Globular glial tauopathy	**+**	**+**	**−**	n.d.	**+**	n.d.	n.d.	Tau (87)
Multiple system atrophy	**+**	**−**	**−**	**−**	**−**	**+**	n.d.	Aβ (26); tau (26); α-syn (25, 36, 38, 88–90)
Dementia with Lewy bodies	**+**	**+**	**−**	n.d.	**−**	**+**	n.d.	α-Syn (38)
Sporadic Parkinson’s disease	**+**	**−**	**−**	n.d.	**−**	**+**	n.d.	α-Syn (38, 91)
Familial Parkinson’s disease	**−**	**+**	**−**	n.d.	n.d.	n.d.	n.d.	n.d.
Creutzfeldt-Jakob disease	**+**	**+**	**+**	n.d.	n.d.	n.d.	**+**	PrP (92–97)
Fatal familial insomnia	**+**	**+**	**−**	n.d.	n.d.	n.d.	**+**	PrP (95, 98)
Kuru	**−**	**−**	**+**	n.d.	n.d.	n.d.	**+**	PrP (99)
Gerstmann-Sträussler-Scheinker disease	**−**	**+**	**−**	n.d.	n.d.	n.d.	**+**	PrP (100, 101)

Abbreviations: α-Syn, α-synuclein; n.d., not done; PrP, prion protein.

Ridley et al. (34) provided the first clues of Aβ prions in the brains of people with DS, but the incubation times in marmosets are much too long for experimental investigations. In contrast, using our rapid cell bioassays, we discovered that the brains of people with DS contain both Aβ and tau prions indistinguishable from those found in AD. Our findings offer an approach to comparative clinical studies of AD and DS. As we learn more about Aβ and tau prions in DS, it may be feasible to develop smaller, shorter, and more informative clinical trials of potential AD treatments (106, 107). Whether advances in human positron emission tomography imaging for both Aβ plaques and NFTs will prove useful in assessing the levels of Aβ and tau prions in the brains of adults with DS who receive putative anti–AD prion therapeutics remains to be established. Last, because the brains of long-lived people with DS exhibit increased prion infectivity, we posit that more molecular studies for people with DS are needed to better understand how age-dependent pathogenic mechanisms in DS cause a divergent prion phenotype from sAD. The outcome of such work may have important implications for developing drugs that are more aptly tailored to improve quality of life for people with DS.

## Materials and Methods

### Study Design.

This case-control study used deidentified human biospecimens from deceased individuals and is exempt from institutional review board approval (i.e., this study is not considered human subject research) in accordance with University of California, San Francisco (UCSF) IRB policy. Samples were collected retrospectively based on availability for distribution and known case criteria. As such, we have followed the STROBE (Strengthening the Reporting of Observational Studies in Epidemiology) reporting guidelines in this article.

### Human Brain Sample Procurement and Processing.

All tissue donors or a proxy provided written or verbal consent to donate postmortem brains for use in biomedical research in accordance with the standards of each institution. Fresh-frozen autopsied brain tissue was procured from several brain biorepositories in the United States and Europe (*SI Appendix*, Tables S1 and S2 include available demographic information of patient donors). Samples were chosen based on available cases and each cohort was age- and sex-matched as a group. Frozen tissues were thawed and weighed to determine the mass in grams. Tissue was mechanically homogenized in nine volumes of cold Dulbecco’s phosphate-buffered saline (DPBS) containing Halt Protease Inhibitor Mixture (1×; Thermo Fisher Scientific) using a handheld probe-tip homogenizer (OMNI International). The homogenate was clarified by centrifugation at 5,000*g* for 5 min at 4 °C, and the supernatants were collected and stored at −80 °C.

### PTA Precipitation of Aβ and Tau in Frozen Brain Samples.

PTA precipitation of human postmortem brain samples was performed as described (26, 40). Briefly, 10% brain homogenate was incubated in 2% sarkosyl and 0.5% benzonase (Sigma) at 37 °C with constant agitation (900 rpm) in an orbital shaker for 2 h. PTA was dissolved in double-distilled water, and the pH was adjusted to 7.0. PTA was added to the solution to a final concentration of 2%, which was then incubated overnight under the same conditions. The sample was centrifuged at 16,000*g* for 75 min at room temperature, and then the supernatant was removed. The resulting pellet was resuspended in DPBS using 10% of the initial starting volume and stored at −80 °C.

### YFP-Tagged HEK293T Cell Bioassay for Measuring Prion Infectivity.

Previously, we developed monoclonal HEK293T cell lines expressing constructs encoding human WT Aβ42 fused with YFP at the N terminus (26). Cell lines expressing human 4R tau (repeat domain) with the mutations P301L and V337M fused with YFP at the C terminus were generated as described previously (37).

To perform the bioassay, 3,000 cells per well (containing 0.1 μg/mL Hoechst 33342) were plated at 70 μL/well onto 384-well plates (Greiner) and incubated for 2 h before treatment with samples. Based on prior work (26), brain extracts (0.03×; 20% final volume) were incubated with Lipofectamine 2000 (1.5% final volume; Thermo Fisher Scientific) and Opti-MEM (78.5% final volume; Thermo Fisher Scientific) for 2 h. Following incubation, samples were plated onto 384-well plates in four replicate wells (10 μL/well). Plates were incubated, and DAPI and FITC channels were imaged every 24 h (five images per well) for 3 d using the GE Healthcare IN Cell Analyzer 6000. Images were analyzed using IN Cell Developer software and custom protocols containing algorithms to detect intracellular aggregates in live cells. Data are presented as integrated total fluorescence intensity per cell.

### Custom Neuropathological Scoring of Aβ Plaque and Tau Tangle Density in Formalin-Fixed Sections.

Standardized, biorepository-provided neuropathological scores (e.g., Braak and Consortium to Establish a Registry for Alzheimer's Disease [CERAD]) were not available for all DS cases procured for this study. Moreover, while such scores reflect the global overview of neuropathological burden across the entire brain, they do not provide the exact neuropathological burden in the specific brain sample of interest. We wanted to quantify the abundance of immunofluorescent-stained histological deposits in formalin-fixed sections from regions adjacent to the frozen samples used for prion infectivity and biochemical measurements. Thus, to obtain a standardized measure of AD neuropathology, we generated our own pathological scores (0, 1, 2, 3, and 4) based on Aβ and tau load in the frontal cortex detected using antibodies targeting Aβ40, Aβ42, and phosphorylated tau, as described in complete detail by Maxwell et al. (62). In brief, 0 = <1 plaque/mm^2^ and <1 mature NFT/mm^2^; 1 = <1 dense-cored plaque but ≥2 total plaques and 1 to 5 NFTs; 2 = ≥1 dense-cored but <2 neuritic plaques and 5 to 12 NFTs; 3 = ≥5 dense-cored and 2 to 15 neuritic plaques and 12 to 25 NFTs; and 4 = ≥15 neuritic plaques and ≥25 mature NFTs. We validated our approach by comparing our scores with the limited Braak and CERAD data that were available and found our metrics to be consistent.

### Immunochemical Protein Quantification in Bulk Tissue Extracts.

To determine the total concentration of soluble APP and tau present in each frontal cortex sample, sandwich enzyme-linked immunosorbent assays (Invitrogen, catalog KHB0051 and KHB0041) were performed on brain homogenate (10% in phosphate-buffered saline [PBS], called “10% BH”) clarified with centrifugation (5,000*g* for 5 min) to remove cell debris and most insoluble proteins. Samples were prepared and stored in low-binding 96-well plates and measured according to manufacturer directions. Protein concentrations were normalized to total brain protein in the clarified homogenate as determined by bicinchoninic acid assay.

Insoluble protein fractions were extracted from brain homogenate by sonicating 10% BH with 75% vol/vol formic acid for 20 min followed by ultracentrifugation at 48,000*g* for 1 h at 4 °C. The supernatant was neutralized with a 20-fold dilution in neutralization buffer (1 M Tris base [NH_2_C(CH_2_OH)_3_] 0.5 M Na_2_HPO_4_⋅7 H_2_O; pH 10.5) and was stored in aliquots at −80 °C until use. To measure concentrations of Aβ40, Aβ42, and insoluble tau species in these extracts, we instead used homogeneous time-resolved fluorescence (HTRF) assays, which were shown to generate more reproducible measures of insoluble (formic acid–soluble) proteins. Total tau (Perkin-Elmer Cisbio 64NTAUPEG), tau phospho-S202/T205 (64TS2PEG), Aβ40 (62B40PEG), and Aβ42 (62B42PEG) HTRF kits were used according to manufacturer protocols.

### Statistical Analysis.

Statistical analyses were performed with GraphPad Prism, version 9. Data are shown as mean ± SD. Comparisons between multiple groups were performed using two-way ANOVA with Tukey’s multiple comparisons test. For two-group comparisons, we used Student’s *t* test. A simple linear regression was performed for all XY scatter plots. A value of *P* < 0.05 was considered significant.

## Supplementary Material

Supplementary File

## Data Availability

All study data are included in the article and/or supporting information.
