# Supplementary material for: Aβ and tau prions feature in the neuropathogenesis of Down syndrome
Source: Proc Natl Acad Sci U S A. 2022 Nov 7;119(46):e2212954119. doi: 10.1073/pnas.2212954119 (PMC9674250; doi:10.1073/pnas.2212954119)
Supplement: Supplementary File [file pnas.2212954119.sapp.pdf]

PNAS

Supporting Information for
Aβ and tau prions feature in the neuropathogenesis of Down syndrome

Carlo Condello[1,2*], Alison M. Maxwell[3], Erika Castillo[1], Atsushi Aoyagi[1,4], Caroline Graff[5,6], Martin Ingelsson[7,8,9], Lars Lannfelt[7], Thomas D. Bird[10,11], C. Dirk Keene[12], William W. Seeley[2,13], Daniel P. Perl[14], Elizabeth Head[15], and Stanley B. Prusiner[1,2,16*]

Carlo Condello and Stanley B. Prusiner
Email: carlo.condello@ucsf.edu and stanley.prusiner@ucsf.edu

This PDF file includes:

Figure S1
Figure S2
Figure S3
Figure S4
Figure S5
Figure S6
Table S1
Table S2
SI References

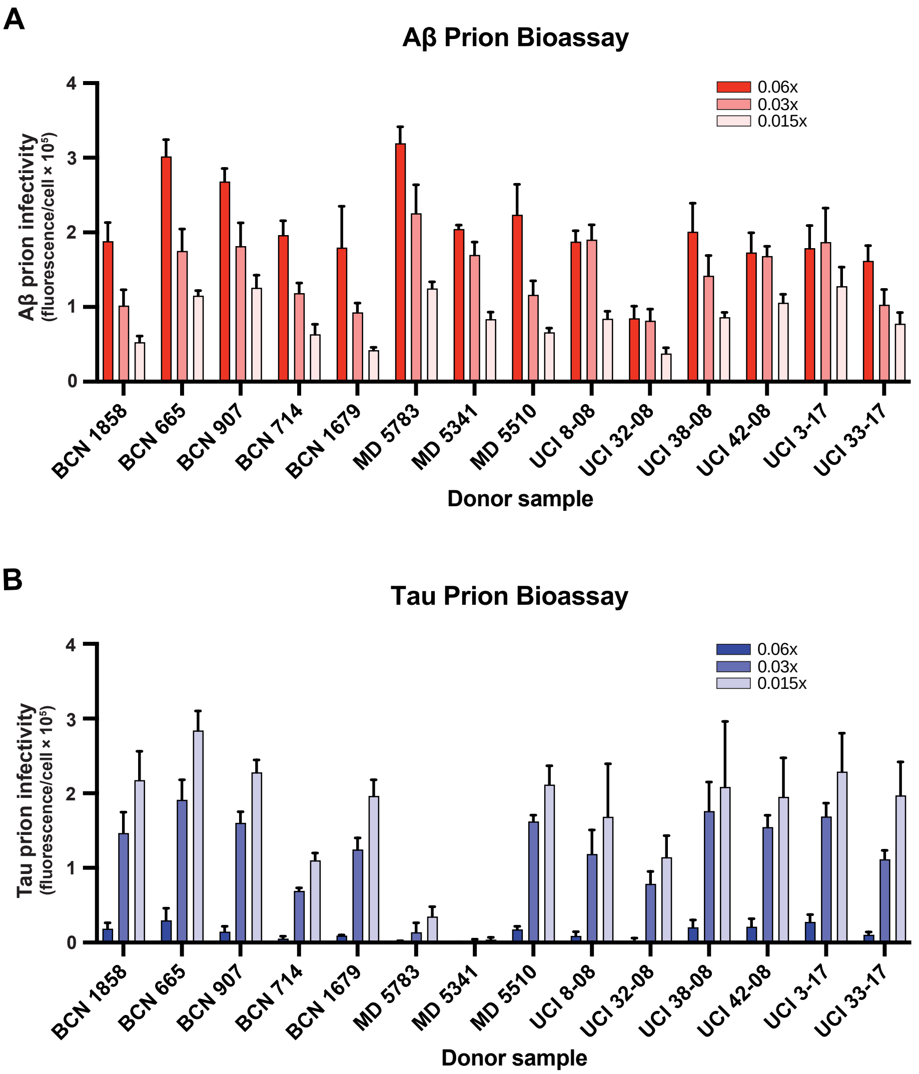

Fig. S1. Titration of human Down syndrome and Alzheimer's disease brain extracts in cell-based bioassays. (*A*, *B*) PTA extracts from Down syndrome (DS) and sporadic Alzheimer's disease (sAD) brain samples were diluted 0.06x, 0.03x, and 0.015x and mixed with lipofectamine prior to infection of HEK293T cells expressing YFP-Aβ42 or tauK18(S)-YFP to measure (*A*) Aβ and (*B*) tau prion infectivity, respectively. Data presented as the mean and standard deviation of four technical replicates per sample.

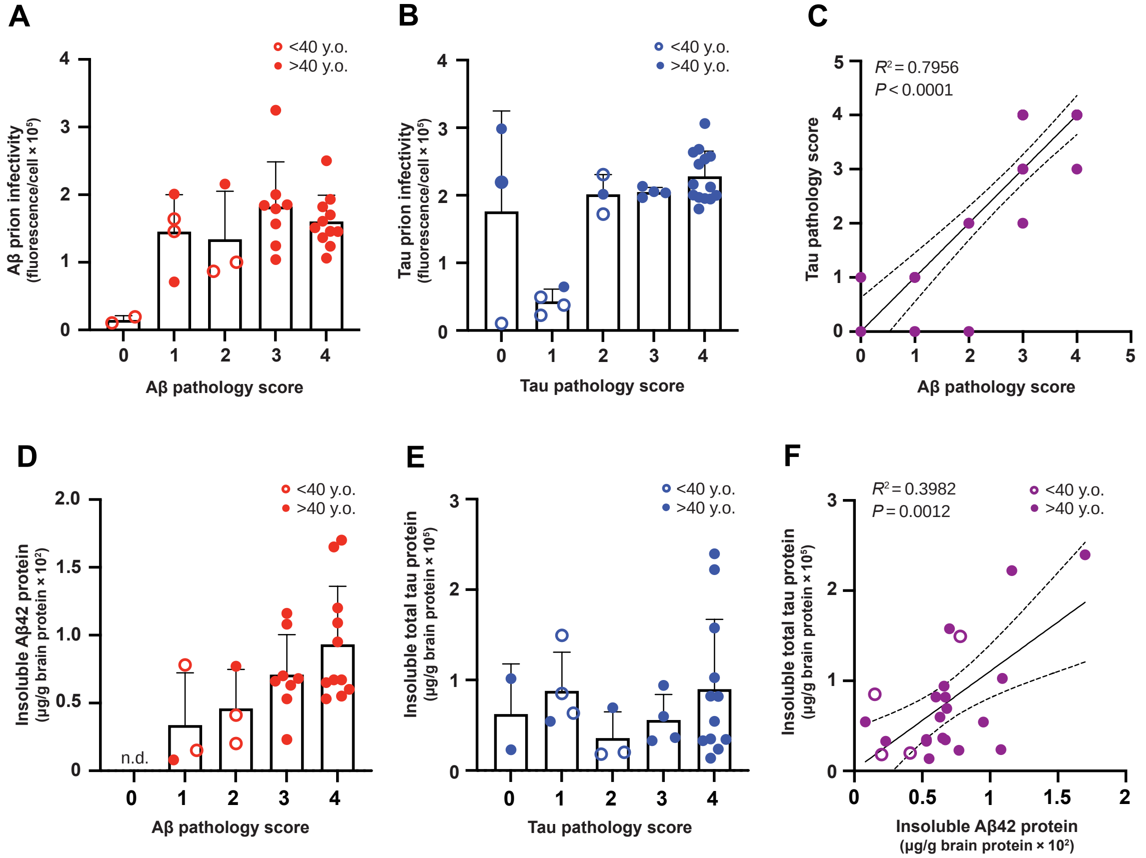

Fig. S2. DS prion abundance corresponds to neuropathological scores of plaques and tangles and immunoassays of insoluble Aβ and tau. We used custom neuropathological scores of Aβ and tau neurofibrillary tangle (NFT) burden measured in immunohistochemically stained formalin-fixed frontal cortex sections from the cohort of donors with DS that was previously reported in Maxwell et. al. (1). For additional details, see Methods. The majority of DS samples had Aβ and tau scores ≥3, corresponding to the presence of many cored and neuritic plaques and NFTs. (*A*) Aβ prion infectivity binned by Aβ plaque pathology score. (*B*) Tau prion infectivity binned by tau NFT pathology score. (*C*) Tau pathology score plotted as a function of Aβ pathology score, and a linear regression was performed. (*D*) Insoluble Aβ42 abundance binned by Aβ plaque pathology score. (*E*) Insoluble total tau abundance binned by tau NFT pathology score. (*F*) Insoluble total tau abundance plotted as a function of insoluble Aβ42 abundance, and a linear regression was performed.

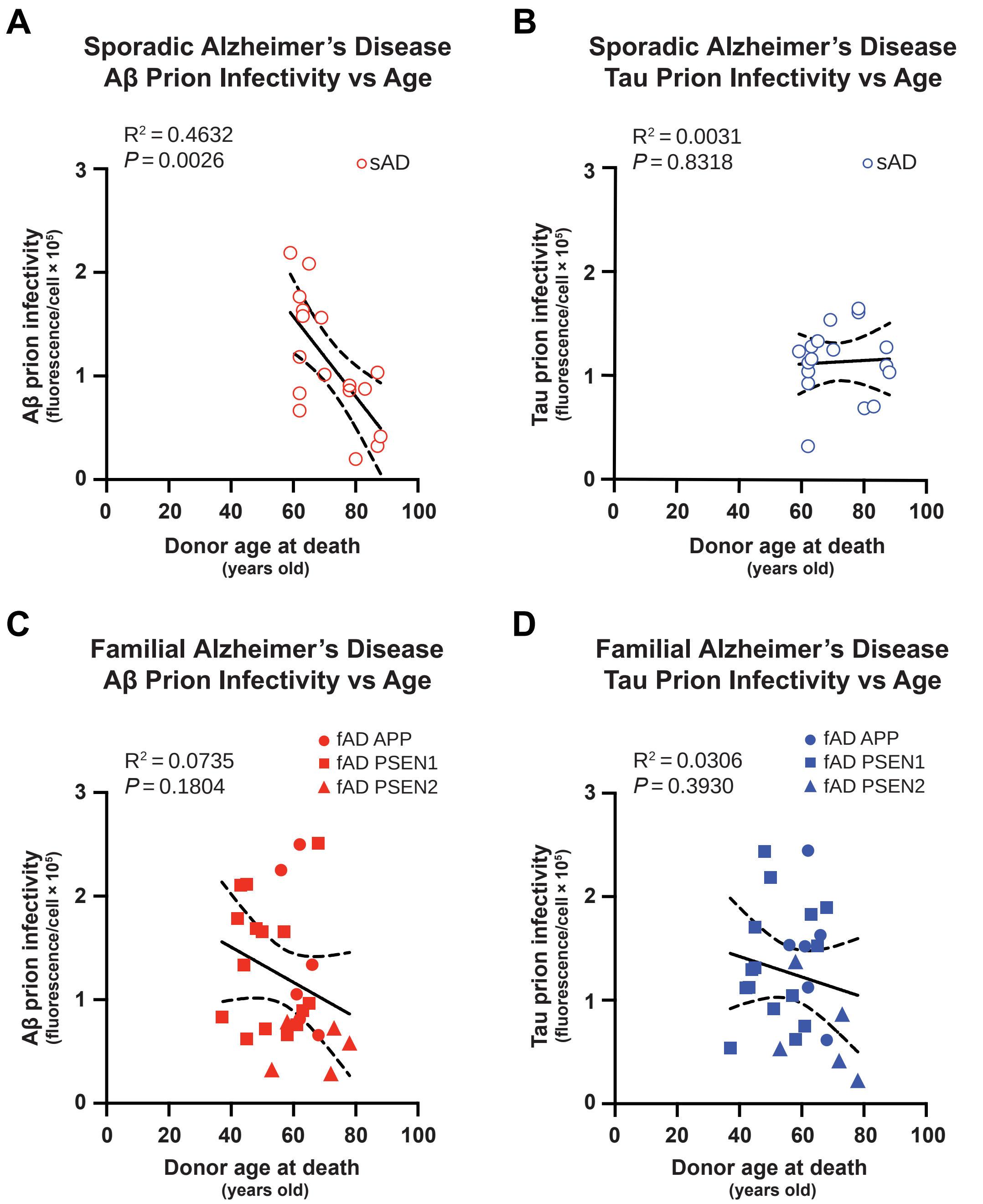

Fig. S3. Relationship of donor age at death and prion abundance in sporadic and familial AD. (*A*, *B*) Aβ and tau prion infectivity in sAD brain samples plotted as a function of donor age at death. (*C*, *D*) Aβ and tau prion infectivity in familial AD (fAD) APP (filled circles), fAD PSEN1 (filled squares), and fAD PSEN2 (filled triangles) brain samples plotted as a function of donor age at death. Linear regression performed in all panels.

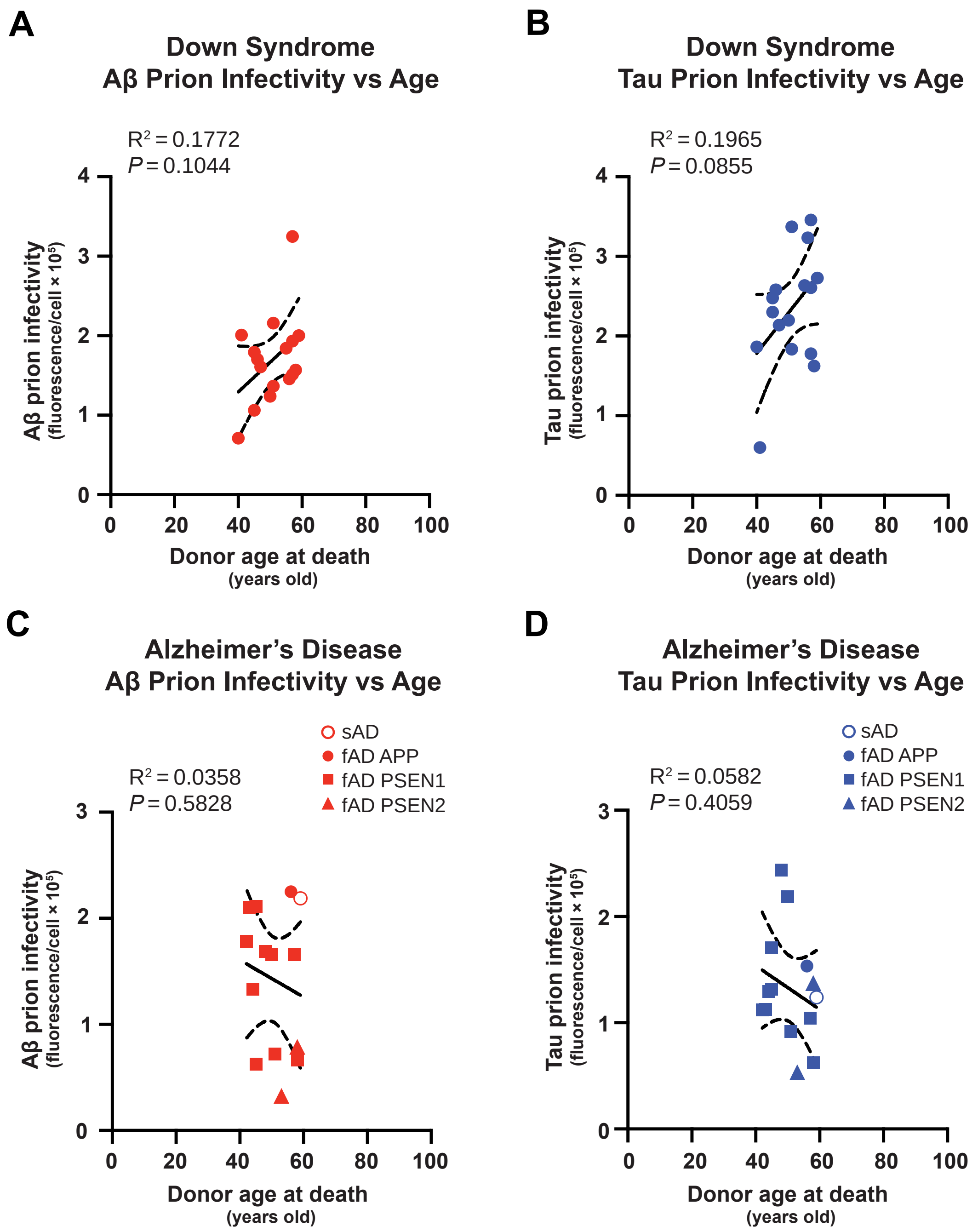

Fig. S4. DS and AD prion abundance plotted for cases with an overlapping donor age range of 40 to 60 years old at death. (*A*, *B*) Aβ and tau prion infectivity in DS brain samples plotted as a function of donor age at death. (*C*, *D*) Aβ and tau prion infectivity in fAD (filled symbols) and sAD (open symbols) brain samples plotted as a function of donor age at death. Linear regression performed in all panels.

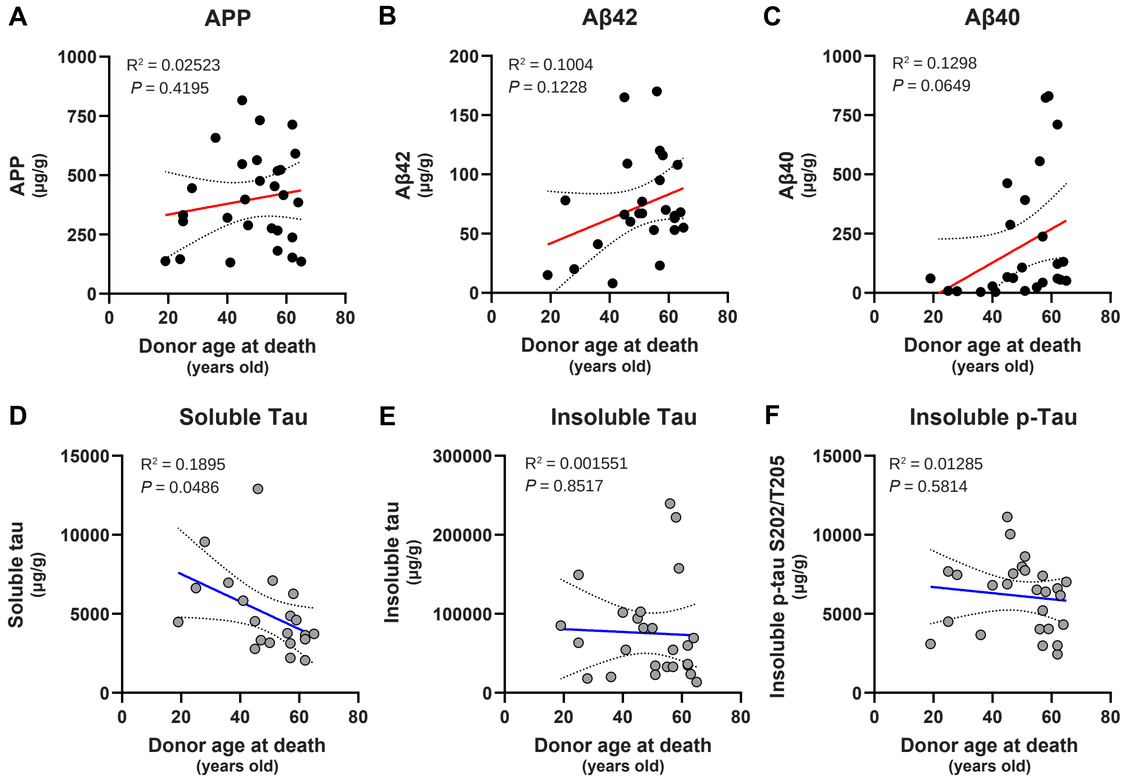

Fig. S5. Immunochemical assays of protein abundance in DS brain samples. (*A*) ELISA measurements of APP protein concentration in soluble brain fractions plotted as a function of donor age at death. (*B*, *C*) HTRF measurements of Aβ42 and Aβ40 concentrations in insoluble brain fractions plotted as a function of donor age at death. (*D*) ELISA measurements of total tau protein concentrations in soluble brain fractions plotted as a function of donor age at death. (*E*, *F*) HTRF measurements of total tau and phospho-S202/T205 tau concentrations in insoluble brain fractions plotted as a function of donor age at death. Linear regression performed in all panels.

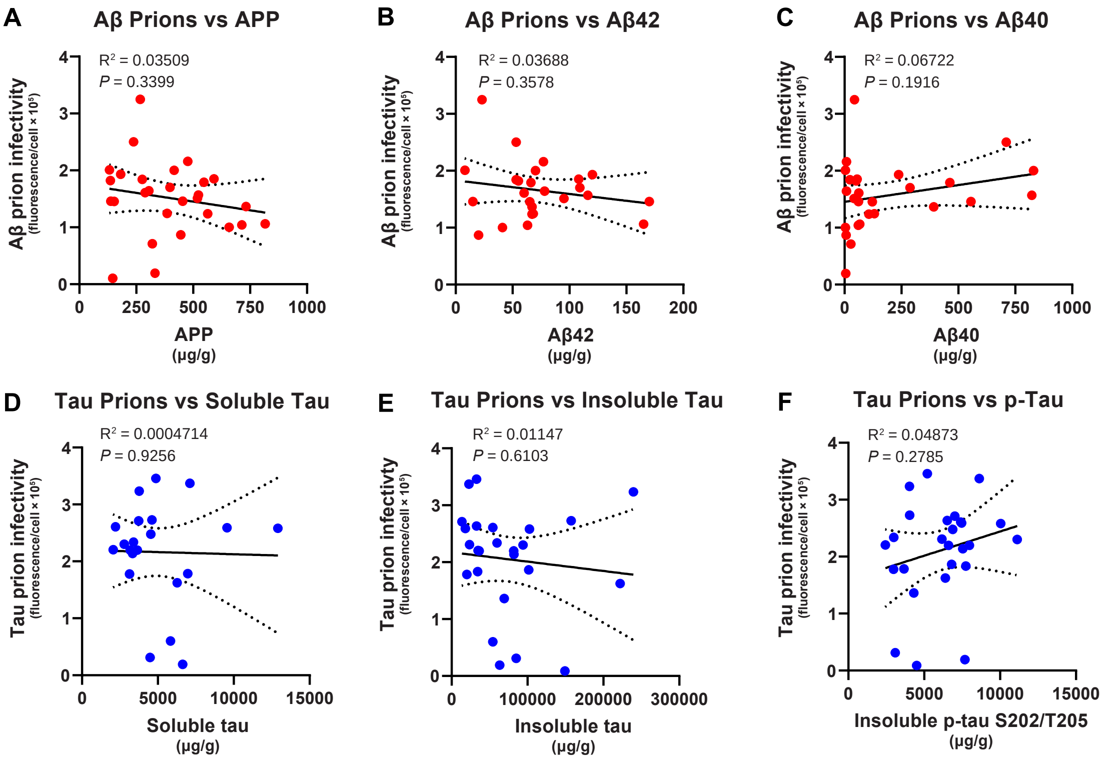

Fig. S6. Correlation of prion infectivity levels with protein abundance in DS brain samples. Immunochemical assays of protein abundance in DS brain samples. (*A*–*C*) Aβ prion infectivity levels plotted as a function of APP, insoluble Aβ42, or insoluble Aβ40 concentration, respectively. (*D*–*F*) Tau prion infectivity levels plotted as a function of soluble total tau, insoluble total tau, or insoluble phospho-S202/T205 tau concentration, respectively. Linear regression performed in all panels.

Table S1. Source of postmortem human DS brain tissue samples. Demographic and clinicopathological data for cases with Down syndrome and age-matched controls.

Cohort	Donor ID#	Age	Sex	NPDX	CERAD Score	Braak Stage	Prion Infectivity		APOE Genotype	Brain Region	Source
							Mean (Fluorescence/cell)				
							Aβ	Tau			
DS	Mia18	24	M	DS	-	-	10517.4	10387.4	3/3	Frontal cortex	University of Miami
DS	MD 5341	25	M	DS	-	-	164045.7	22432.0	3/3	Frontal cortex	University of Maryland
DS	MD 4870	51	F	DS	-	-	136564.9	179783.6	2/3	Frontal cortex	University of Maryland
DS	MD 4904	40	M	DS	-	-	71043.9	219329.7	3/3	Frontal cortex	University of Maryland
DS	MD 5713	25	M	DS	-	-	19365.5	37428.2	2/3	Frontal cortex	University of Maryland
DS	MD 5600	57	F	DS	-	-	324715.3	306222.9	3/3	Frontal cortex	University of Maryland
DS	MD 5386	64	M	DS	-	-	124471.0	201766.5	3/4	Frontal cortex	University of Maryland
DS	MD 5277	19	M	DS	-	-	145977.3	49129.4	3/4	Frontal cortex	University of Maryland
DS	MD 6151	57	M	DS	-	VI	193228.4	216381.0	3/3	Frontal cortex	University of Maryland
DS	MD 4530	47	F	DS	-	-	160955.4	196871.0	3/4	Frontal cortex	University of Maryland

Cohort	Donor ID#	Age	Sex	NPDX	CERAD Score	Braak Stage	Prion Infectivity Mean (Fluorescence/cell)		APOE Genotype	Brain Region	Source
							Aβ	Tau			
DS	MD 4785	55	F	DS	-	-	184294.0	205480.1	3/3	Frontal cortex	University of Maryland
DS	MD 4335	28	M	DS	-	IV	86964.3	230664.2	4/4	Frontal cortex	University of Maryland
DS	MD 4659	46	F	DS	-	-	170006.7	257720.8	3/4	Frontal cortex	University of Maryland
DS	MD 5783	41	M	DS	-	VI	200947.7	64518.4	3/4	Frontal cortex	University of Maryland
DS	BCN 665	59	F	DS	C2	VI	200267.9	246127.9	3/4	Frontal cortex	IDIBAPS (Barcelona)
DS	BCN 714	36	F	DS	C2	II	100221.7	172328.8	3/3	Frontal cortex	IDIBAPS (Barcelona)
DS	BCN 907	63	M	DS	C3	VI	185071.2	253341.2	3/3	Frontal cortex	IDIBAPS (Barcelona)
DS	BCN 1335	62	F	DS	C3	VI	250195.6	212427.8	3/4	Frontal cortex	IDIBAPS (Barcelona)
DS	BCN 1469	62	M	DS	C3	VI	145553.5	203759.2	3/4	Frontal cortex	IDIBAPS (Barcelona)
DS	UCI 30-05	57	F	DS	C3	VI	151304.4	263981.9	3/3	Frontal cortex	University of California, Irvine
DS	UCI 10-13	56	M	DS	C3	VI	145692.1	268189.9	4/4	Frontal cortex	University of California, Irvine
DS	UCI 39-17	58	M	DS	C3	VI	156991.0	195424.7	3/4	Frontal cortex	University of California, Irvine

Cohort	Donor ID#	Age	Sex	NPDX	CERAD Score	Braak Stage	Prion Infectivity Mean (Fluorescence/cell)		APOE Genotype	Brain Region	Source
							Aβ	Tau			
DS	UCI 31-10	62	F	DS	C3	VI	104181.0	213442.8	3/3	Frontal cortex	University of California, Irvine
DS	UCI 29-06	45	F	DS	C3	VI	106270.2	194510.4	3/3	Frontal cortex	University of California, Irvine
DS	UCI 5-15	51	F	DS	C3	III	215680.4	298710.1	3/3	Frontal cortex	University of California, Irvine
DS	UCI 1-11	45	F	DS	C3	VI	179305.3	196788.5	3/3	Frontal cortex	University of California, Irvine
DS	UCI 21-14	50	M	DS	C3	VI	123793.0	199903.9	2/3	Frontal cortex	University of California, Irvine
DS	MD 5510	65	M	DS	-	-	182136.2	200480.6	3/3	Frontal cortex	University of Maryland
Control	RB 18-01	35	M	NCI	-	-	5203.3	885.2	-	Frontal cortex	Uniformed Services University of the Health Sciences
Control	RB 18-16	42	M	NCI	-	-	3009.0	6998.3	-	Frontal cortex	Uniformed Services University of the Health Sciences
Control	RB 19-12	66	M	NCI	-	-	2983.0	9240.5	-	Frontal cortex	Uniformed Services University of the Health Sciences
Control	RB 16-23	58	M	NCI	-	-	11765.1	371.9	-	Frontal cortex	Uniformed Services University of the Health Sciences

Cohort	Donor ID#	Age	Sex	NPDX	CERAD Score	Braak Stage	Prion Infectivity Mean (Fluorescence/cell)		APOE Genotype	Brain Region	Source
							Aβ	Tau			
Control	RB 16-22	51	M	NCI	-	-	630.6	1948.2	-	Frontal cortex	Uniformed Services University of the Health Sciences
Control	RB 16-19	41	M	NCI	-	-	3176.2	1341.8	-	Frontal cortex	Uniformed Services University of the Health Sciences
Control	RB 18-29	70	M	NCI	-	-	24610.9	22953.2	-	Frontal cortex	Uniformed Services University of the Health Sciences
Control	RB 16-16	52	M	NCI	-	-	7015.7	876.1	-	Frontal cortex	Uniformed Services University of the Health Sciences
Control	RB 18-06	27	M	NCI	-	-	1646.5	1950.8	-	Frontal cortex	Uniformed Services University of the Health Sciences
Control	RB 18-15	67	M	NCI	-	-	5074.6	745.7	-	Frontal cortex	Uniformed Services University of the Health Sciences
Control	RB 18-09	62	M	NCI	-	-	12924.9	3835.2	-	Frontal cortex	Uniformed Services University of the Health Sciences
Control	RB 18-25	44	M	NCI	-	-	6636.0	8354.2	-	Frontal cortex	Uniformed Services University of the Health Sciences

Cohort	Donor ID#	Age	Sex	NPDX	CERAD Score	Braak Stage	Prion Infectivity		APOE Genotype	Brain Region	Source
							Mean (Fluorescence/cell)				
							Aβ	Tau			
Control	RB 19-13	35	M	NCI	-	-	5782.0	1388.7	-	Frontal cortex	Uniformed Services University of the Health Sciences
Control	RB 18-14	37	M	NCI	-	-	5936.0	797.5	-	Frontal cortex	Uniformed Services University of the Health Sciences

Abbreviations: DS, Down syndrome; NCI, no cognitive impairment; NPDX, neuropathological diagnosis; PMI, postmortem interval; CERAD, Consortium to Establish a Registry for Alzheimer's Disease; APOE, apolipoprotein E.

Table S2. Source of postmortem human AD brain tissue samples. Demographic and clinicopathological data for cases with familial and sporadic Alzheimer's disease and age-matched controls.

Cohort	Donor ID#	Sex	Age	NPDX	Mutation	CERAD Score	Braak Stage	Prion Infectivity Mean (Fluorescence/cell)		APOE Genotype	Brain Region	Source
								Aβ	Tau			
sAD	P1952	F	82	AD	-	C3	V-VI	87574.9	71119.5	-	Frontal cortex	UCSF NDBB
sAD	P2217	M	63	AD	-	C3	VI	163400.0	128582.0	3/4	Temporal cortex	UCSF NDBB
sAD	P2273	M	63	AD	-	C3	VI	157781.0	116578.0	3/3	Temporal cortex	UCSF NDBB
sAD	P2287	F	69	AD	-	C3	VI	156471.0	154386.0	3/3	Temporal cortex	UCSF NDBB
sAD	P2312.10	M	59	AD	-	C3	VI	218990.0	123934.0	4/4	Temporal cortex	UCSF NDBB
sAD	P2330.11	M	70	AD	-	C3	VI	101308.0	125670.0	3/4	Temporal cortex	UCSF NDBB
sAD	P2350.11	F	65	AD	-	C3	VI	208337.0	133892.0	2/3	Temporal cortex	UCSF NDBB
sAD	BBN_6072	M	78	AD,CAA, SVD	-	C2	III	90763.8	161592.0	3/4	Temporal cortex	University of Manchester (UK)
sAD	BBN_3416	M	87	AD,CAA, SVD	-	C2	IV	103453.0	110250.0	3/4	Temporal cortex	University of Manchester (UK)

Cohort	Donor ID#	Sex	Age	NPDX	Mutation	CERAD Score	Braak Stage	Prion Infectivity Mean (Fluorescence/cell)		APOE Genotype	Brain Region	Source
								Aβ	Tau			
sAD	BBN_15592	M	62	AD,LBD	-	C3	-	176745.0	93067.6	3/4	Temporal cortex	University of Manchester (UK)
sAD	#10	F	85	AD	-	C2	V-VI	83436.2	32677.4	-	Temporal cortex	Uppsala Unversity (Sweden)
sAD	12-1181	M	62	AD	-	C3	VI	66640.2	104679.7	-	Frontal cortex	University of Washington (Seattle)
sAD	07-0997	F	87	AD	-	C3	VI	32384.1	128180.3	3/3	Occipital Cortex	University of Washington (Seattle)
sAD	2412.12	M	88	AD	-	C3	VI	41659.2	104035.4	3/4	Parietal cortex	UCSF NDBB
sAD	2376.11	M	80	AD	-	C3	VI	19901.9	69475.6	3/3	Parietal cortex	UCSF NDBB
sAD	P1959	M	61	AD	-	C3	V-VI	118498	113219.9	-	Frontal cortex	UCSF NDBB
sAD	P2478	M	78	AD	-	C3	V	86123.6	165391.9	-	Temporal cortex	UCSF NDBB
fAD APP	#2	F	61	AD, CAA	KM670/671NL APP	C3	V-VI	105313.0	152202.0	3/4	Temporal cortex	Uppsala Unversity (Sweden)

Cohort	Donor ID#	Sex	Age	NPDX	Mutation	CERAD Score	Braak Stage	Prion Infectivity		APOE Genotype	Brain Region	Source
								Mean (Fluorescence/cell)				
								Aβ	Tau			
fAD APP	6901	M	62	AD	KM670/671NL APP	C3	VI	249857.0	244544.0	3/3	Frontal cortex	Karolinska Institutet (Sweden)
fAD APP	493	M	68	AD	KM670/671NL APP	C3	-	65952.0	61586.0	2/3	Parietal cortex	Karolinska Institutet (Sweden)
fAD APP	39794	M	66	AD	KM670/671NL APP	C3	VI	133822.0	162773.0	2/3	Frontal cortex	Karolinska Institutet (Sweden)
fAD APP	7795	M	56	AD	KM670/671NL APP	C3	VI	225026.0	153367.0	4/4	Frontal cortex	Karolinska Institutet (Sweden)
fAD APP	14096	F	62	AD	KM670/671NL APP	C3	VI	81449.5	112489.0	3/3	Frontal cortex	Karolinska Institutet (Sweden)
fAD PSEN	BBN_13829	M	42	AD	Δ4 PSEN1	C3	VI	178324.0	111941.0	3/3	Temporal cortex	King's College London (UK)

Cohort	Donor ID#	Sex	Age	NPDX	Mutation	CERAD Score	Braak Stage	Prion Infectivity Mean (Fluorescence/cell)		APOE Genotype	Brain Region	Source
								Aβ	Tau			
fAD PSEN	BBN_3246	F	57	AD	M139V PSEN1	C3	VI	165718.0	104424.0	3/3	Temporal cortex	University of Manchester (UK)
fAD PSEN	3206	M	45	AD	I143T PSEN1	C3	VI	211418.0	170413.0	3/3	Frontal cortex	Karolinska Institutet (Sweden)
fAD PSEN	12908	F	43	AD	I143T PSEN1	C3	VI	210281.0	112727.0	3/4	Frontal cortex	Karolinska Institutet (Sweden)
fAD PSEN	00-0700	M	37	AD	I143T PSEN1	C3	VI	83235.2	53602.2	3/3	Frontal cortex	University of Washington (Seattle)
fAD PSEN	4602	F	50	AD	I143T PSEN1	C3	VI	165621.0	218704.0	3/4	Frontal cortex	Karolinska Institutet (Sweden)

Cohort	Donor ID#	Sex	Age	NPDX	Mutation	CERAD Score	Braak Stage	Prion Infectivity		APOE Genotype	Brain Region	Source
								Mean (Fluorescence/cell)				
								Aβ	Tau			
fAD PSEN	2598	F	48	AD	I143T PSEN1	C3	VI	168997.0	243669.0	3/4	Frontal cortex	Karolinska Institutet (Sweden)
fAD PSEN	1104	M	68	AD	H163Y PSEN1	C3	V-VI	250990.0	189349.0	3/4	Frontal cortex	Karolinska Institutet (Sweden)
fAD PSEN	8715	M	63	AD	H163Y PSEN1	C3	V-VI	89638.3	183206.0	2/4	Frontal cortex	Karolinska Institutet (Sweden)
fAD PSEN	12829	M	45	AD	G209V PSEN1	C3	VI	62281.3	131365.0	3/3	Frontal cortex	University of Washington (Seattle)
fAD PSEN	25044	F	51	AD	G209V PSEN1	C3	VI	72232.7	91817.4	2/3	Frontal cortex	University of Washington (Seattle)

Cohort	Donor ID#	Sex	Age	NPDX	Mutation	CERAD Score	Braak Stage	Prion Infectivity Mean (Fluorescence/cell)		APOE Genotype	Brain Region	Source
								Aβ	Tau			
fAD PSEN	01-0164	M	58	AD	G209V PSEN1	C3	V	66485.9	62452.8	2/3	Frontal cortex	University of Washington (Seattle)
fAD PSEN	02-0621	F	44	AD	A260V PSEN1	C3	VI	133364.0	129629.0	3/3	Frontal cortex	University of Washington (Seattle)
fAD PSEN	BBN_13813	F	65	AD	E280G PSEN1	C3	VI	96323.7	152366.0	3/4	Temporal cortex	King's College London (UK)
fAD PSEN	00-0281	M	61	AD	A431E PSEN1	C3	VI	75891.9	74949.8	3/3	Frontal cortex	University of Washington (Seattle)
fAD PSEN	12540	M	72	AD	N141I PSEN2	C3	VI	28987.9	41728.4	3/4	Frontal cortex	University of Washington (Seattle)

Cohort	Donor ID#	Sex	Age	NPDX	Mutation	CERAD Score	Braak Stage	Prion Infectivity Mean (Fluorescence/cell)		APOE Genotype	Brain Region	Source
								Aβ	Tau			
fAD PSEN	23156	F	78	AD	N141I PSEN2	C3	V	58334.4	22640.7	2/3	Frontal cortex	University of Washington (Seattle)
fAD PSEN	26111	F	53	AD	N141I PSEN2	C3	V	32669.1	53298.1	3/4	Frontal cortex	University of Washington (Seattle)
fAD PSEN	11-0929	M	58	AD	N141I PSEN2	C3	VI	78907.4	137379.0	3/3	Frontal cortex	University of Washington (Seattle)
fAD PSEN	12-0370	M	73	AD	N141I PSEN2	C3	VI	72891.8	86654.1	-	Frontal cortex	University of Washington (Seattle)
Control	HCTMI_15_001	M	62	NCI	-	-	-	10823.7	8710.97	-	Putamen	University of Miami
Control	HCTQJ_15_002	F	67	NCI	-	-	-	11257.7	2463.3	-	Putamen	University of Miami

Cohort	Donor ID#	Sex	Age	NPDX	Mutation	CERAD Score	Braak Stage	Prion Infectivity Mean (Fluorescence/cell)		APOE Genotype	Brain Region	Source
								Aβ	Tau			
Control	HCTNU_15_0 08	M	59	NCI	-	-	-	9712.2	8442.7	-	Putamen	University of Miami
Control	HCTLT_15_0 09	M	65	NCI	-	-	-	7980.0	3918.0	-	Putamen	University of Miami
Control	P78/06	F	68	NCI	-	-	-	6240.9	8486.5	-	Frontal cortex	University College London
Control	PDC023	-	-	NCI	-	-	-	3563.0	3058.6	-	Frontal cortex	UKPD Brain Bank
Control	P2207	F	81	NCI	-	C0	II	2431.8	8625.9	3/3	Temporal cortex	UCSF NDBB
Control	P2594	F	86	NCI	-	C0	II	8993.2	12192.8	-	Temporal cortex	UCSF NDBB
Control	#23	F	88	NCI	-	C0	II	9175.8	20084.3	-	Frontal cortex	Uppsala University
Control	#24	M	63	NCI	-	C0	0	4208.7	16558.6	-	Frontal cortex	Uppsala University

Abbreviations: sAD, sporadic AD; fAD, familial AD; CAA, cerebral amyloid angiopathy; SVD, small vessel disease; LBD, Lewy body dementia; APP, amyloid precursor protein; PSEN, presenilin; NCI, no cognitive impairment; NPDX, neuropathological diagnosis; CERAD, Consortium to Establish a Registry for Alzheimer's Disease; APOE, apolipoprotein E; UCSF NDBB, UCSF Neurodegenerative Disease Brain Bank.

SI References

1. A. M. Maxwell *et al.*, Emergence of distinct and heterogeneous strains of amyloid beta with advanced Alzheimer's disease pathology in Down syndrome. *Acta Neuropathol Commun* **9**, 201 (2021).
